# Identification of a novel lipid metabolism-related gene signature for predicting colorectal cancer survival

**DOI:** 10.3389/fgene.2022.989327

**Published:** 2022-09-06

**Authors:** Yanpeng Huang, Jinming Zhou, Haibin Zhong, Ning Xie, Fei-Ran Zhang, Zhanmin Zhang

**Affiliations:** ^1^ Department of General Surgery, The First Affiliated Hospital of Shantou University Medical College, Shantou, Guangdong, China; ^2^ GenePlus-Beijing, Beijing, China; ^3^ Department of Cancer Center, The First Affiliated Hospital of Nanchang University, Nanchang, Jiangxi, China

**Keywords:** colorectal cancer, lipid metabolism, signature, prognosis, biomarkers

## Abstract

Colorectal cancer (CRC) is a common malignant tumor worldwide. Lipid metabolism is a prerequisite for the growth, proliferation and invasion of cancer cells. However, the lipid metabolism-related gene signature and its underlying molecular mechanisms remain unclear. The aim of this study was to establish a lipid metabolism signature risk model for survival prediction in CRC and to investigate the effect of gene signature on the immune microenvironment. Lipid metabolism-mediated genes (LMGs) were obtained from the Molecular Signatures Database. The consensus molecular subtypes were established using “ConsensusClusterPlus” based on LMGs and the cancer genome atlas (TCGA) data. The risk model was established using univariate and multivariate Cox regression with TCGA database and independently validated in the international cancer genome consortium (ICGC) datasets. Immune infiltration in the risk model was developed using CIBERSORT and xCell analyses. A total of 267 differentially expressed genes (DEGs) were identified between subtype 1 and subtype 2 from consensus molecular subtypes, including 153 upregulated DEGs and 114 downregulated DEGs. 21 DEGs associated with overall survival (OS) were selected using univariate Cox regression analysis. Furthermore, a prognostic risk model was constructed using the risk coefficients and gene expression of eleven-gene signature. Patients with a high-risk score had poorer OS compared with patients in the low-risk score group (*p* = 3.36e-07) in the TCGA cohort and the validationdatasets (*p* = 4.03e-05). Analysis of immune infiltration identified multiple T cells were associated with better prognosis in the low-risk group, including Th2 cells (*p* = 0.0208), regulatory T cells (*p* = 0.0425), and gammadelta T cells (*p* = 0.0112). A nomogram integrating the risk model and clinical characteristics was further developed to predict the prognosis of patients with CRC. In conclusion, our study revealed that the expression of lipid-metabolism genes were correlated with the immune microenvironment. The eleven-gene signature might be useful for prediction the prognosis of CRC patients.

## Introduction

Colorectal cancer (CRC) is the third most common malignant cancer worldwide (Johdi and Sukor, 2020), which will project a total of 2.2 million new cases and 1.1 million deaths by 2030 (Douaiher et al., 2017). Therefore, early intervention for primary CRC contributes to clinical benefit outcomes (Koncina et al., 2020). At present, the main treatment methods for CRC are tumor resection, radiotherapy, chemotherapy, anti-angiogenic therapy, targeted therapy and immunotherapy (Rejhova et al., 2018; Piawah and Venook, 2019; Dariya et al., 2020; Johdi and Sukor, 2020). Although a certain degree of success has been achieved with these treatments, several significant challenges remain to be addressed. (Biller and Schrag, 2021). However, the etiology and molecular mechanisms of CRC are still unclear. Previous studies have identified prognostic and predictive molecular biomarkers for CRC based on DNA, RNA, or proteins, such as APC, VEGF-1 (Clarke, 2005; Das et al., 2017; Koncina et al., 2020). Liu et al. (2020) identified two risky (TIMP1 and LZTS3) and five protective prognostic genes (AXIN2, CXCL1, ITLN1, CPT2, and CLDN23) which provided more evidence for further application of novel diagnostic and prognostic biomarkers in CRC. A prognostic signature consisting of nine genes was established with good performance for the prediction of survival in CRC patients (Chen et al., 2019). Moreover, novel potential prognostic biomarkers still need to be explored for patient risk stratification and for the choice of best treatment options. Therefore, it is necessary to screen novel molecular therapeutic targets to improve the survival rate of CRC patients.

Lipids are essential components of biological membranes and are signaling molecules involved in cellular activities (Bian et al., 2021). Lipid metabolism plays an important role in maintaining of cellular homeostasis (Rohrig and Schulze, 2016; Bian et al., 2021). Numerous studies have demonstrated that lipid metabolism was involved in the progression, recurrence and tumor microenvironment (TME) of CRC (Lin et al., 2021). Dysregulation of lipid metabolism occurs in multiple cancers, including CRC (Dias et al., 2019; Haffa et al., 2019). Numerous bioactive secondary messengers trigger the activation of RAS, phosphoinositide 3-kinases (PI3Ks) and other signaling pathways to promote tumorigenesis (Yang et al., 2019; Moore et al., 2020). A previous study has shown that a total of 13 metabolites, including glycerophospholipids, were associated with a reduced risk of recurrence in CRC patients (Ose et al., 2021). Blocking metabolic reprogramming of tumor cells in obese mice improves anti-tumor immunity by impairing CD8^+^ T cell infiltration in the tumor microenvironment (Ringel et al., 2020). However, studies focusing on the characterization and risk signatures of lipid metabolism-related genes remain limited.

**FIGURE 1:**
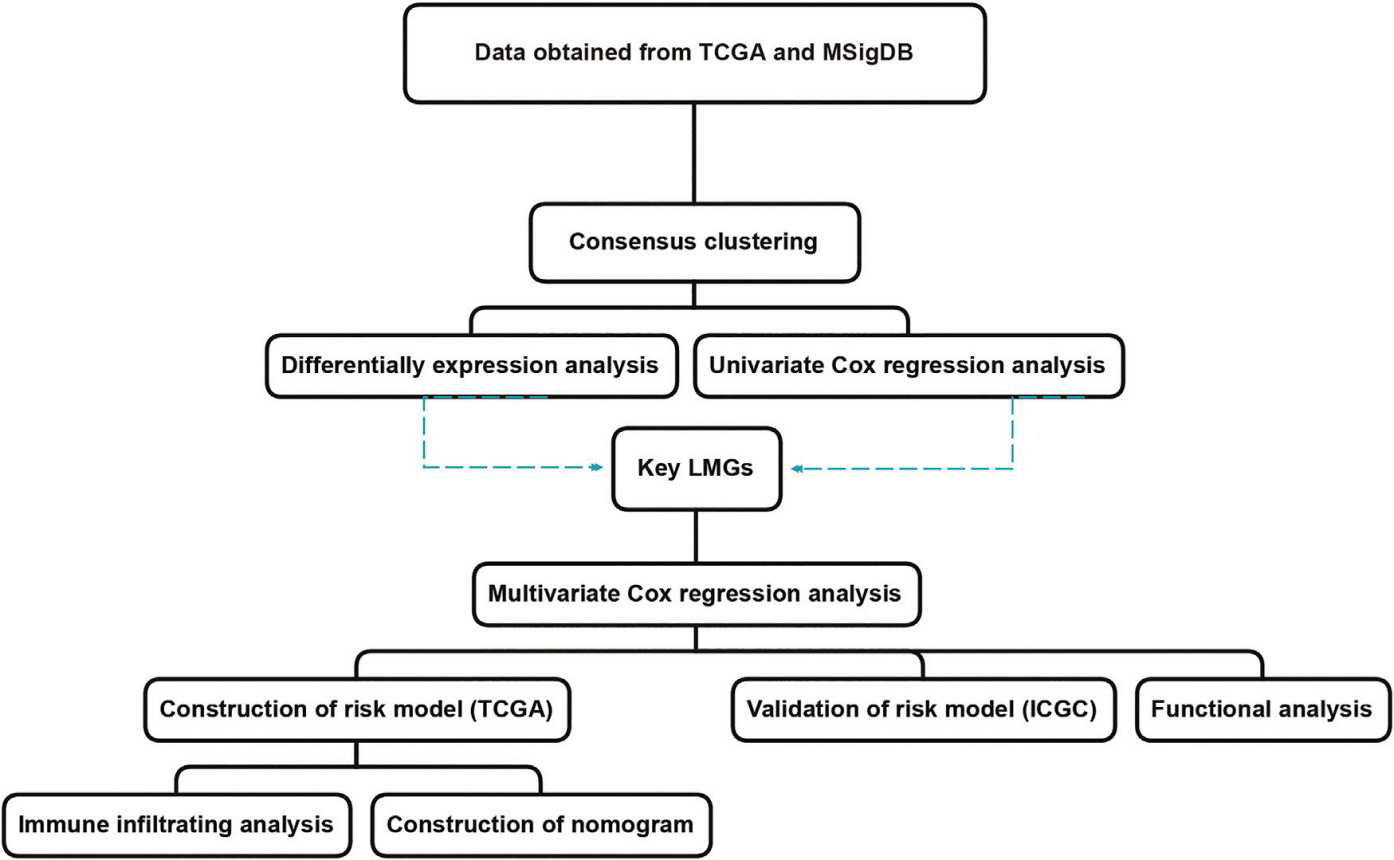
The workflow of lipid metabolism-related signature identification. TCGA: The Cancer Genome Atlas. MSigDB: Molecular Signatures Database. ICGC: International Cancer Genome Consortium. LMGs: Lipid metabolism-associated genes.

The aim of this study was to screen genes closely related to the prognosis of CRC using two published datasets. The 11-gene signature risk model provided a reference to distinguish high-risk groups in CRC patients with poor prognosis (Wang et al., 2021b). The workflow was illustrated in Figure 1.

## Materials and methods

### Data collection and preparation

RNA sequencing (RNA-seq) data and clinical features were retrieved from The Cancer Genome Atlas (TCGA) database (404 samples, TCGA-COAD, https://portal.gdc.cancer.gov/). Furthermore, the ICGC datasets (302 samples, COAD-US, https://dcc.icgc.org/releases/current/Projects/) were used to validate the risk model. RPKM is a widely used method for normalizing RNA-seq gene expression (Guo et al., 2013). All data were analyzed using RPKM expression profiles, including differentially expressed genes (DEGs), consensus clustering, analysis of tumor-infiltrating immune cells and univariate and multivariate Cox regression analyses. Lipid metabolism-associated genes (LMGs) were obtained from the Molecular Signature Database (MSigDB, http://gsea-msigdb.org). We selected the following keywords to select Lipid metabolism-associated genes (LMGs), including “lipid,” “lipid metabolism,” “metabolism of lipid,” “fat metabolism,” “fatty acid metabolism,” “metabolism of fat.” A total of 744 LMGs were selected from MSigDB and provided in the Supplementary Table S1.

### Consensus clustering and DEGs analysis

Consensus analysis was performed using the “ConsensusClusterPlus” R package to assign patients with COAD into different clusters in the TCGA dataset. Subsequently, the DEGs were obtained between clusters with *p* < 0.05 and |log2 (fold change)| > 0.5 as the threshold using “limma” package in R software. The volcano plot was visualized by “ggplot2” R package.

### Immune infiltration

Immune and stromal scores were calculated to evaluate cell infiltration levels in CRC. Estimation of Stromal and Immune cells in Malignant Tumor tissues using Expression data (ESTIMATE) algorithm (https://bioinformatics.mdanderson.org/estimate/) was adopted to measure stromal level (stromal scores), immunocyte infiltration degree (immune scores), and tumor purity using “estimate” R package (Li et al., 2020b; Guo et al., 2020).

The differences in immune infiltration subtypes were analyzed between the high- and low-risk groups using two-way ANOVA analysis. Furthermore, xCell algorithm was used to estimate 64 immune and stromal cell types from transcriptome data using “xCell” R package (Aran et al., 2017).

### Functional enrichment analysis

To explore the signaling pathway enrichment of overlapping genes, the Gene Ontology (GO) term and Kyoto Encyclopedia of Genes and Genomes (KEGG) pathway enrichment analyses were performed based on the online platform KOBAS (http://kobas.cbi.pku.edu.cn/index.php) with *p* < 0.05. The visualization was performed using “ggplot2” R package (Ito and Murphy, 2013).

### Construction of prognostic gene model

Univariate Cox regression analysis was used to identify the lipid metabolism-related genes associated with prognosis of CRC with *p* < 0.05. The multivariate Cox regression analysis was performed to screen independent prognostic signature. Risk score for the signature was evaluated as following algorithm: Riskscore = Coef_gene1_*expression_gene1_ + Coef_gene2_*expression_gene2_ + Coef_gene3_*expression_gene3_ + ...... + Coef_genen_*expression_genen_ (where “Coef” and “expression” are respectively the coefficient and RNA relative expression value, “gene” represents each selected gene range from 1 to n) (Wang et al., 2020b). Briefly, firstly, a robust likelihood-based survival modeling approach was used to narrow the number of genes from 21 key LMGs and the best genes were selected for the prognostic model using “survminer” and “survival” R package (Wang et al., 2021c). Secondly, multiple Cox regression analysis was performed to establish prognostic risk model using “survival” R package with a parameter of “direction = “both” (Wang et al., 2020b). Thirdly, the prognostic risk model of each sample was calculated with coefficient value of multiple Cox regression analysis using the following: Risk score = (−0.376743) × GGT5 + (−0.572140) × ASAH1 + (−0.484800) × HMGCL + (0.670476) × CD36 + (0.733487) × DPM2 + (−0.463117) × ACOX1 + (0.506670) × ANGPTL4 + (0.434523) × INSR + (−0.504028) × ADIPOQ + (0.615982) × ALDH1A3 + (−0.195776) × MMP1. The median parameter of risk score was 1.012238. The ROC curve was plotted using “survivalROC” R package with a parameter of “method = “KM” (Heagerty et al., 2000). The “pheatmap” R package (version 1.0.2, https://cran.r-project.org/web/packages/pheatmap/index.html) to show the heat map. The nomogram was formed to estimate the overall survival (OS) of CRC by using “rms” and “survival” package in R. The regression coefficients of the regression model was transformed into scores and plotted as a nomogram for prediction of prognosis. Moreover, the calibration curves were used to evaluate the precision of nomogram for the probability of actual occurrence versus prediction, with Hosmer-Lemeshow fit goodness test (Zhou et al., 2019).

## Results

### Identification of two molecular subtypes and DEGs

**FIGURE 2:**
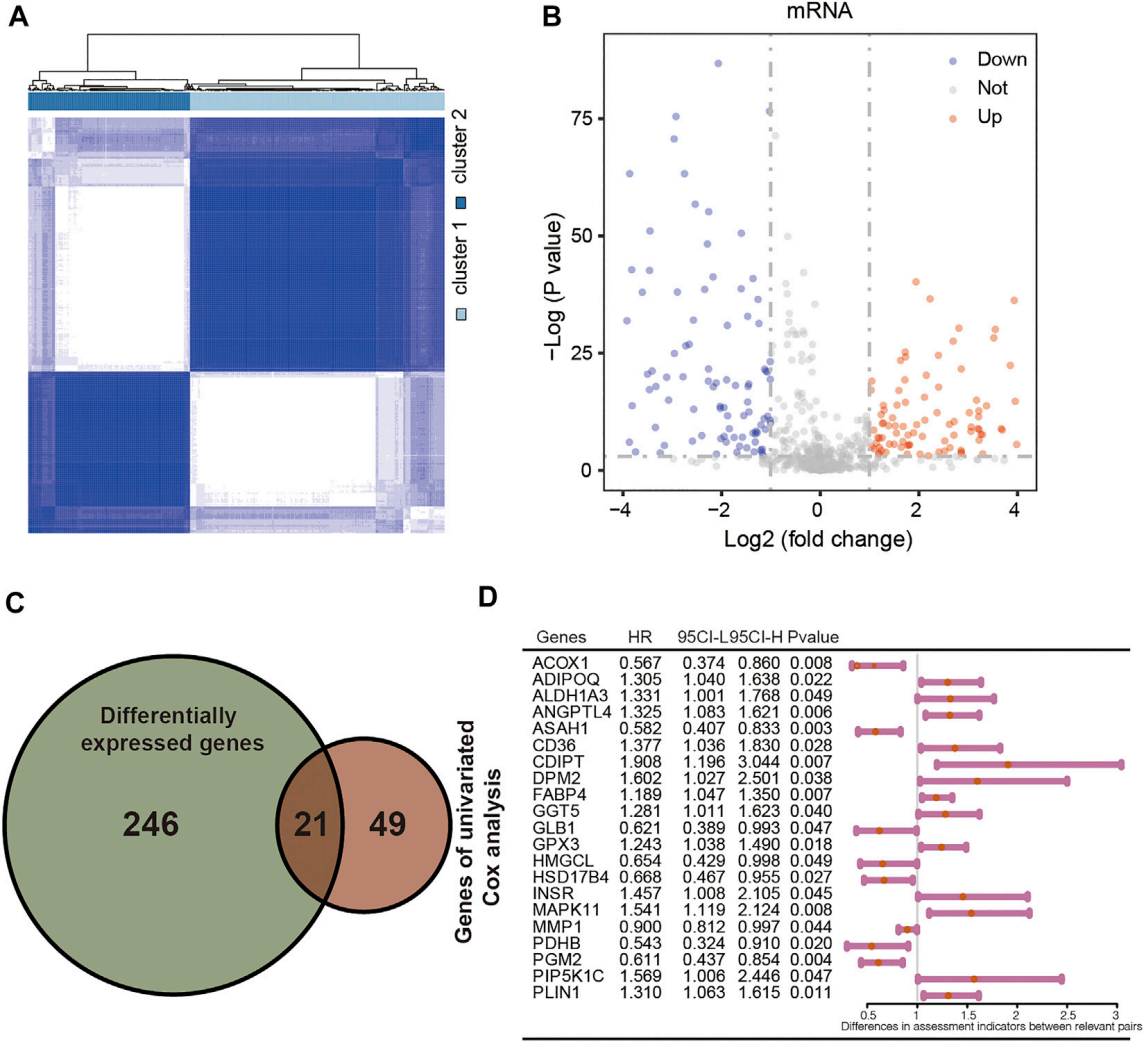
Identification of differentially expressed LMGs with consensus molecular subtypes and univariate Cox regression analysis. **(A)** Molecular subtype discrimination in TCGA. **(B)** Differentially expressed LMGs between subtype 1 and subtype 2. **(C)** Venn diagram showing 21 key LMGs (the intersection of the differentially expressed LMGs and the overall survival associated LMGs using the univariate Cox regression analysis). **(D)** Univariated cox proportional hazards analysis of key LMGs. HR, hazard ratio; CI, confidence interval. LMGs: Lipid metabolism-associated genes.

A total of 744 LMGs were selected from MSigDB in Methods or Results and provided the terms in the Supplementary Table S1. Consensus clustering was conducted to divide the samples of 404 patients with CRC into subtypes. Optimal clustering stability was identified with *K* = 2 as the cut-off criterion (Supplementary Figures S1A,B). These samples were divided into two molecular subtypes including 251 samples in subtype 1 and 153 samples in subtype 2 (Figure 2A). To explore the dysregulated genes between two molecular subtypes, the differentially expressed genes (DEGs) visualized in volcano plot were screened by using “limma” R package (Ritchie et al., 2015). A total of 267 DEGs were identified with *p* < 0.05 and |Log2 (fold change)| > 0.5, including 153 upregulated DEGs and 114 downregulated DEGs (Figure 2B). Furthermore, univariate Cox regression analysis was performed to calculate the hazard ratio (HR) for OS. A total of 21 genes had a statistically significant effect on OS in CRC with overlapping DEGs (Figure 2C). The HR values of the 21 genes were calculated and shown in Figure 2D as potential molecular targets.

### Establishment of the lipid metabolism-related gene risk signature

**FIGURE 3:**
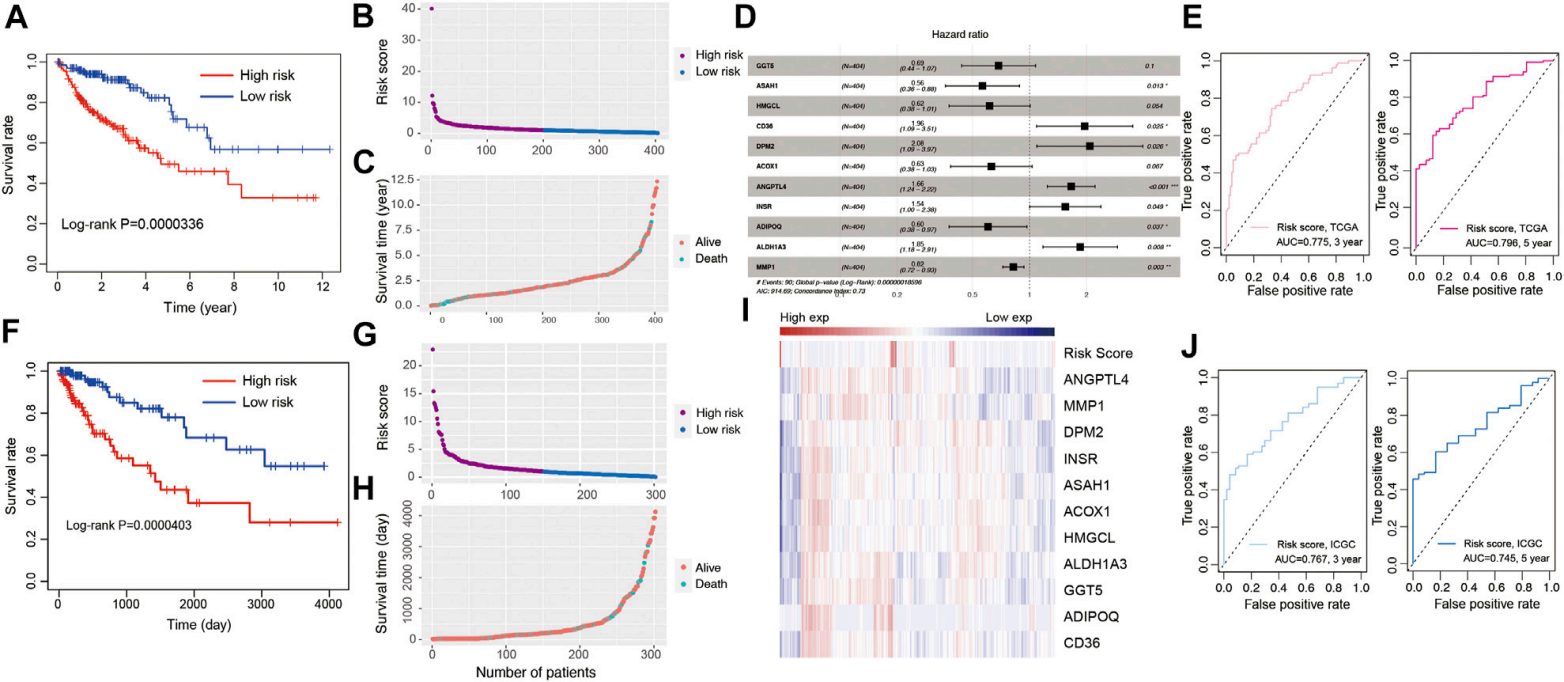
Development of risk model based on the 11 LMGs signature of CRC patients with the TCGA training cohort and ICGC validation database. **(A)** Kaplan-Meier survival plot for overall survival based on risk score of LMGs signature in TCGA cohort. **(B)** Risk score of CRC patients in high- and low risk groups in TCGA cohort. **(C)** Distribution of time under survival curve and survival status of each patient in TCGA cohort. **(D)** Multivariate Cox regression analysis of LMGs signature characteristics TCGA cohort. **(E)** ROC curve for 3 and 5 years overall survival TCGA cohort. **(F)** Kaplan-Meier survival plot showing overall survival using risk score of LMGs signature in ICGC validation database. **(G)** Risk score of CRC patients in risk groups in ICGC validation database. **(H)** Distribution of survival time and survival status of each patient in ICGC validation database. **(I)** The heatmap showing the gene expression of 11 LMGs signature and risk score in ICGC validation database. **(J)** ROC curve for 3 and 5 years overall survival in ICGC validation database. LMGs: Lipid metabolism-associated genes.

To establish a lipid metabolism-related gene signature, the predictive value of the risk score model was explored using the TCGA-COAD database as the training set. A total of 21 genes were intersection of those identified by differentially expression analysis and univariate Cox regression analysis. After that, a robust likelihood-based survival modeling approach was used to narrow the number of genes and select the best genes for the prognostic model using “survminer” and “survival” R package (Wang et al., 2021c). Finally, a total of 11 genes were screened to construct the risk model by using the multivariate Cox regression analysis with a parameter of “direction = both.” To evaluate the survival risk of patients with CRC, a prognostic risk model was constructed using risk coefficients and gene expression as described in previous studies (Zhang et al., 2020; Liu et al., 2021). Each patient’s risk score in the training was calculated using a mathematical algorithm. Firstly, we calculated the coefficient value of each genes using multiple Cox regression analysis. Secondly, risk score was calculated using coefficient value and expression of each gene. The mathematical algorithm in this study was described in the methods and materials section. The median risk score was 1.012238. By the median value of the risk score, all patients were divided into high-risk and low-risk groups according to the median of risk score. Patients with high-risk scores had higher mortality rate and poorer prognosis (Figure 3A, *p* = 3.36e-07). Supplementary Table S2 showed the number of patients along the overall survival and risk scores in TCGA and ICGC database. The risk score rank distribution of patients with CRC were shown in Figure 3B. The scatter represented the survival status and the time under the survival curve in each patient in Figure 3C. The risk scores between high- and low-risk groups in TCGA and ICGC database were added in Supplementary Table S3. The risk signature in the multivariate Cox model was illustrated in the forest plot (Figure 3D). The area under the curve (AUC) values of the ROC curve for 3-years and 5-years OS were 0.775 and 0.796, respectively (Figure 3E). The novel 11-gene prognostic signature was validated in 302 patients with CRC using the ICGC database. Survival analysis indicated that patients in the high-risk group had poorer prognosis (Figure 3F, *p* = 4.03e-05, Supplementary Table S2). The risk score rank distribution and survival status in each patient was shown in Figures 3G,H. The expression of the 11-gene signature and the risk score were visualized in Figure 3I. ROC analysis revealed that the risk model showed a good prediction accuracy, with the AUC of 0.767 (3-years overall survival) and AUC of 0.745 (5-years overall survival) (Figure 3J).

### Functional enrichment analysis

**FIGURE 4:**
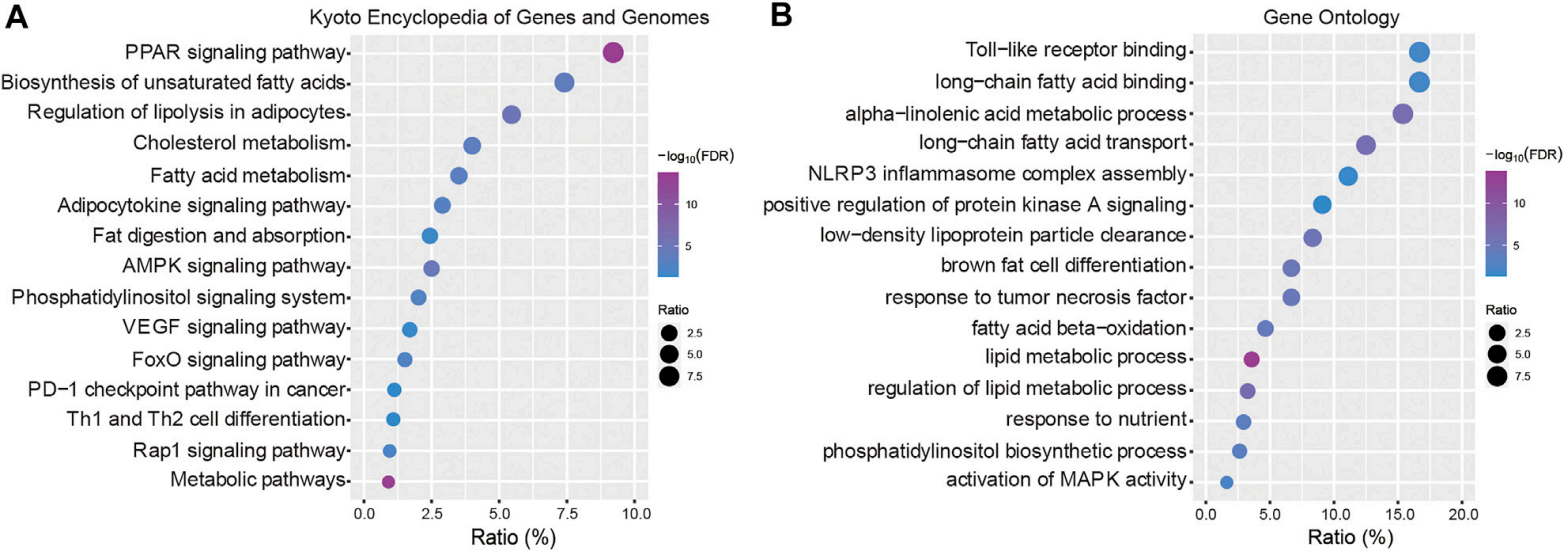
Functional enrichment analysis. **(A)** Kyoto Encyclopedia of Genes and Genomes (KEGG) pathways enriched in the key LMGs. **(B)** Gene Ontology (GO) terms. “Ratio” presents the number of genes in our data/the number of all genes in terms or pathways. LMGs: Lipid metabolism-associated genes. The false discovery rate (FDR) < 0.05 was considered as threshold. The lower boundary value for the -log10(FDR) is 1.301 and the upper boundary value is infinite.

To explore the biological functions of the 21 genes, the Kyoto Encyclopedia of Genes and Genomes (KEGG) pathways and gene ontology (GO) terms were analyzed using KOBAS tools (http://kobas.cbi.pku.edu.cn). In the present study, these dysregulated DEGs were enriched in fatty acid metabolism and multiple cancer-related pathways including VEGF signaling, the PD-1 checkpoint pathway in cancer, the FoxO signaling pathway and Th1 and Th2 cell differentiation (Figure 4A). According to the GO term analysis, we found biological process related to nutrients, including lipid metabolic processes, fatty acid processes and toll-like receptor binding (Figure 4B).

### Assessment of cell scores in tumor microenvironment

**FIGURE 5:**
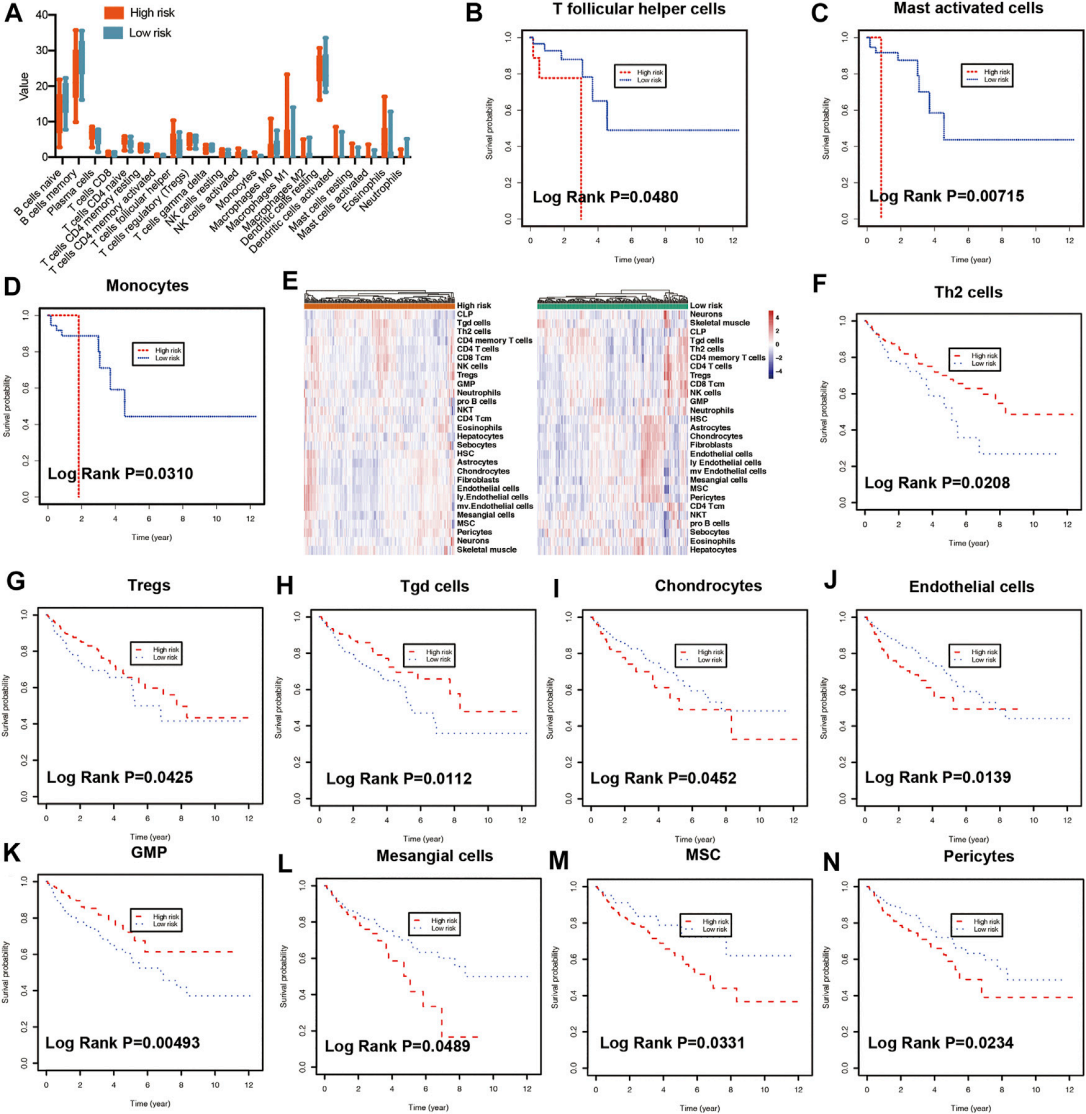
Immune characteristics of risk groups. **(A)** Different immune cell levels between high-risk and low-risk groups using CIBERSORT analysis. Kaplan-Meier plot showing prognostic values of the risk signature between high- and low-risk groups in multiple immune cells using CIBERSORT analysis, including T follicular helper cells **(B)**, mast activated cells **(C)**, and monocytes **(D)**. **(E)** The heatmap showing the abundance scores of immune cells in risk groups using xCell analysis. Kaplan-Meier plot illustrating prognostic values of the risk signature between high- and low-risk groups using xCell analysis in different immune cells, including Th2 cells **(F)**, Tregs **(G)**, Tgd cells **(H)**, Chondrocytes **(I)**, Endothelial cells **(J)**, GMP **(K)**, Mesangial cells **(L)**, MSC **(M)**, Pericytes **(N)**. Th2: T helper 2 cells, Treg: Regulatory T cells. Tgd cells: gamma delta T cells. GMP: Granulocyte-macrophage progenitor, MSC: mesenchymal stem cells.

To predict the cell scores in the tumor microenvironment, the CIBERSORT and xCell algorithms were used to perform cell type enrichment analysis in CRC. The relative infiltration levels of various immune cell subsets were quantified using CIBERSORT algorithms (Chong et al., 2021). In the present study, a consistent result was observed in the risk score stratification (Figure 5A). Furthermore, several kinds of immune cells with high infiltration levels were significantly correlated with poorer prognosis of patients, including T follicular helper cells (Figure 5B, *p* = 0.048), mast activated cells (Figure 5C, *p* = 0.00715) and monocytes (Figure 5D, *p* = 0.031). In addition, the xCell method was used to estimate the abundance scores of 64 immune cell types using lipid metabolism-associated gene expression data. The stratification of abundance scores between the high- and low-risk groups for each patient was demonstrated in Figure 5E. Moreover, the correlation between immune cells and OS was assessed by using Kaplan-Meier survival with log-rank test. Our results demonstrated that the following cells with high abundance scores were associated with better prognosis, including Th2 cells (*p* = 0.0208, Figure 5F), regulatory T cells (Tregs) (*p* = 0.0425, Figure 5G), gamma delta T cells (Tgd cells) (*p* = 0.0112, Figure 5H) and GMP (*p* = 0.00493, Figure 5K). While other immune cells with high abundance scores, including chondrocytes (*p* = 0.0452, Figure 5I), endothelial cells (*p* = 0.0139, Figure 5J), mesangial cells (*p* = 0.0489, Figure 5L), mesenchymal stem cells (MSC, *p* = 0.0331, Figure 5M), Pericytes (*p* = 0.0234, Figure 5N), related to poorer prognosis.

### Predictive nomogram model of independent clinical factors

**FIGURE 6:**
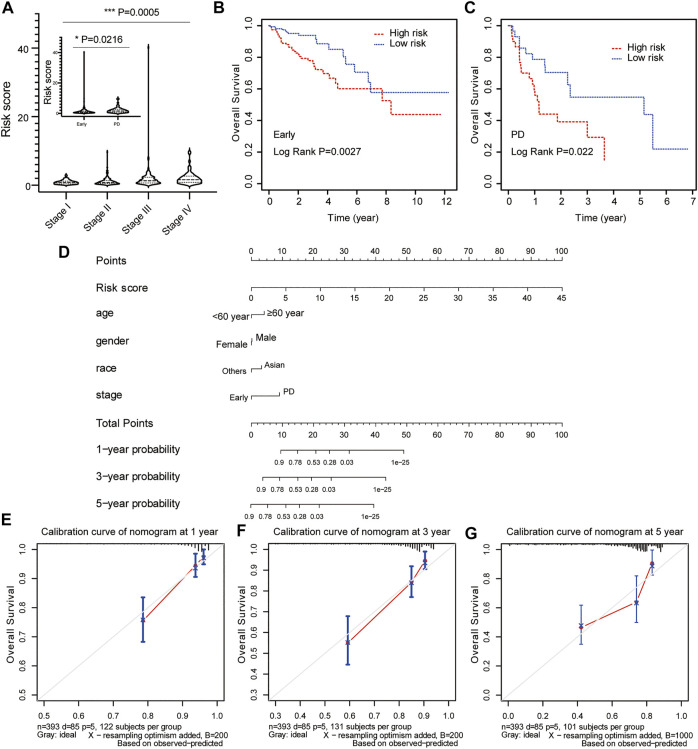
Construction and calibration of nomogram for prognostic prediction in CRC patients. **(A)** The association between risk score and clinical stage. The prognostic value of the risk signature in patients with early/locally advanced CRC (stage I–III) **(B)** and advanced CRC (stage IV) **(C)**. **(D)** The predictive nomogram based on risk score and clinical parameters for overall survival prediction at 1, 3, and 5 years. Calibration curve of the nomogram at 1 year **(E)**, 3 years **(F)**, and 5 years **(G)**.

To evaluate the predictive value of risk model based on clinical features, the relationship between risk score and clinicopathological variables (age, gender, race, and stage) was calculated with the Student’s *t-*test and One-Way ANOVA test. Our results showed that the risk score contributed to different roles in the subgroups, including stage (Figure 6A, *p* = 0.0005), age (Supplementary Figure S2A, *p* = 0.1003), gender (Supplementary Figure S2B, *p* = 0.1932), race (Supplementary Figure S2C, *p* = 0.3185). Patients with early-stage CRC had a lower risk score compared with patients with advanced CRC (Figure 6A, *p* = 0.0216). The Kaplan-Meier survival curve showed that patients in the high-risk group had a dramatically shorter OS than those in the low-risk group in both early-stage CRC (Figure 6B, *p* = 0.0027) and advanced CRC (Figure 6C, *p* = 0.022).

Risk score model status was incorporated into a nomogram model to predict the probability of overall survival at 1-, 3- and 5-years model. The point scale at the top of the nomogram model showed the score of every indicator, including age, gender, race, stage and risk score. All the points of each indicator were summed to estimate probability of OS at 1-, 3- and 5-years in nomogram plot (Figure 6D). Furthermore, the calibration curve was constructed to evaluate the performance of nomogram model. The C-index was 0.761 for OS prediction in training data, showing fair agreements between the nomogram prediction and actual observation for the 1-, 3- and 5-years OS (Figures 6E–G).

## Discussion

Colorectal cancer (CRC) is the second leading cause of death worldwide since 2020 (https://www.who.int/news-room/fact-sheets/detail/cancer). Previous studies showed prognostic models contributed to clinical decision and precision medicine (Zhang et al., 2020; Mohammed et al., 2021). The patients were divided into high- and low-risk groups for prediction of overall survival according to the risk stratification in the prognostic models (Lin et al., 2021). It is critical for improvement of the personalized therapies and the quality of life.

Intensively proliferating cancer cells need multiple metabolic patterns to get enough energy for new biomass synthesis (Warburg et al., 1927). A previous study demonstrated that lipid metabolism played critical roles in the main determinants of tumor progression (Bleve et al., 2020). Cancer cells show alterations of lipid metabolism, which lead to dysregulation of energy homeostasis, disruption of gene expression and signaling pathway (Huang and Freter, 2015; Pakiet et al., 2019). Numerous lipid molecules involved in lipid metabolism were considered as potential biomarkers, including serum polyunsaturated fatty acid metabolites (Zhang et al., 2017), cerotic acid (26:0) (Mika et al., 2017), γ-linolenic acid (18:3 n-6) (Kondo et al., 2011), and 12-keto-leukotriene B4 (Savari et al., 2014). However, the development of clinically useful lipid biomarkers requires a consistent research methodology. The aim of this study is to investigate the association between lipid metabolism and survival, and construct a lipid metabolism-based risk signature to improve the accuracy of prognosis prediction for survival in CRC patients.

Different colorectal cancer classification systems were identified by using microsatellite instability and highly expressed mesenchymal genes. However, these systems failed to incorporate with other subtypes (Wilkerson and Hayes, 2010). Consensus molecular subtypes were clustering algorithms with resampling and network-based approaches (Wilkerson and Hayes, 2010). Consensus molecular subtypes revealed prognostic value in metastatic colorectal cancer (Borelli et al., 2021). Here, the consensus molecular subtypes were established using “ConsensusClusterPlus” package in R software (Wilkerson and Hayes, 2010). In head and neck squamous cell carcinoma, the molecular features of different subtypes were evaluated for potentially effective therapeutic agents (Zhang et al., 2021). In gastric cancer, consensus molecular subtypes were associated with immune infiltration for prediction of survival (Yu et al., 2021a). In metastatic colorectal cancer, consensus molecular analysis demonstrated that the consensus molecular subtype 2 was the predominant subtype in left-sided and associated with the best outcome from the addition of bevacizumab to first-line chemotherapy (Mooi et al., 2018). Our results proved the molecular diversity of lipid metabolism-associated genes and provided different classification strategy for treatment allocation in CRC. A total of 267 differentially expressed genes were screened between cluster 1 subtype and cluster 2 subtype according to consensus molecular analysis. Furthermore, risk model was established using novel 11-gene signature from DEGs and revealed that high-risk group had poorer prognosis (*p* = 3.36e-07). The risk model was validated to divide patients into high- and low-risk groups for OS prediction (*p* = 4.03e-05). Similarly, a novel 4 gene prognostic signature revealed dramatically influence of clinical utility with risk model in colorectal cancer (Ahluwalia et al., 2019).

Numerous evidences from preclinical and clinical data support that the cancer stem cells (CSCs) are responsible for tumor recurrence (Peitzsch et al., 2017; Clarke, 2019). Lipid metabolism has been reported as potential target in bulk and CSCs, including CRC (Li et al., 2017; Choi et al., 2019). A previous study showed that blocking stearoyl-CoA desaturase 1 (SCD1) expression or function inhibited the survival of CSCs, but not bulk colorectal cancer cells *in vitro* and *in vivo* (Yu et al., 2021b). Stem colorectal cancer cells contained a distinctive lipid profile, with higher free MUFA and lower free SFA levels than bulk colorectal cancer cells through lipidomic profiling (Choi et al., 2019). Another study identified eicosapentaenoic acid, which decreased the cell number of the overall population of bulk colorectal cancer cells, but not of the stem colorectal cancer cells. Our results screened 11-gene signature of lipid metabolism for prediction of overall survival in colorectal cancer. Further in-depth studies are also warranted to elucidate the role of 11-gene signature on the behavior in bulk and stem colorectal cancer cells.

To further investigate the effect of nomogram on the predictive ability of survival, novel significant molecular signatures were screened for predicting OS in patients with CRC. The immune related signature showed better stratification and more precise immunotherapy in patients with CRC (Li et al., 2020a). A 13-gene metabolic signature was constructed to explore the association between metabolism and the immune microenvironment for prognostic prediction in stomach adenocarcinoma (Ye et al., 2021). In this study, a 11-lipid metabolism-related gene signature was established and showed improved prediction of OS for CRC patients, including GGT5, ASAH1, HMGCL, CD36, DPM2, ACOX1, ANGPTL4, INSR, ADIPOQ, ALDH1A3, and MMP1. GGT5 (Gamma-Glutamyltransferase 5) is a member of the gamma-glutamyl transpeptidase gene family involved in glutathione metabolism (Wickham et al., 2011). GGT5 was associated with immune cell infiltration and might be a potential immunological therapeutic target in gastric cancer (Wang et al., 2022). ASAH1 (N-Acylsphingosine Amidohydrolase 1) encodes a member of the acid ceramidase family of proteins and is involved in glycosphingolipid metabolism (Li et al., 1999). ASAH1 was used to build a risk model to reflect the dysregulated metabolic microenvironment in gastric cancer (Wen et al., 2020). HMGCL (3-Hydroxy-3-Methylglutaryl-CoA Lyase) is a mitochondrial enzyme and associated with HMG-CoA lyase deficiency (Menao et al., 2009). HMGCL was potential tumor suppressor gene and associated with poor prognosis in clear cell renal cell carcinoma (Cui et al., 2019). In colon adenocarcinoma, HMGCL was screened as prognosis-related metabolic gene using risk model analysis (Zhao et al., 2021). CD36 (CD36 Molecule) is a transmembrane glycoprotein that participates in adipose energy storage, and gut fat absorption (Smith et al., 2008; Tran et al., 2011; Wang and Li, 2019). A previous study demonstrated that ablation of CD36-mediated FA uptake attenuated tumor progression (Wang and Li, 2019). Moreover, CD36 was found to promote sterile inflammation and activate the protumor ability of tumor-associated immune cells (Wang and Li, 2019). DPM2 (Dolichyl-Phosphate Mannosyltransferase Subunit 2) serves as a donor of mannosyl residues on the lumenal side of the endoplasmic reticulum (Radenkovic et al., 2021). A total of 19 lipid metabolism-related genes were used to establish the risk predictive score model as a potential prognostic indicator of gastric cancer, including DPM2 (Wei et al., 2021). Similarly, our result showed that DPM2 was a lipid metabolism-related prognostic gene in colorectal cancer using risk model analysis. ACOX1 (Acyl-CoA Oxidase 1) is the first enzyme of the fatty acid beta-oxidation pathway (Ferdinandusse et al., 2007). A total of 10 key genes involved in the esophageal cancer progression were used to constructe a risk model for prediction of survival, including ACOX1 (Wang et al., 2021a). The higher expression levels of ACOX1 were related to poorer prognosis in esophageal squamous cell carcinoma (*p* = 0.0051), but better prognosis in esophageal adenocarcinoma (*p* = 0.01). Our results revealed that the high expression of ACOX1 had poorer prognosis in colorectal cancer. The correlation between ACOX1 and overall survival of color or rectal cancer will be investigated in the future. ANGPTL4 (Angiopoietin Like 4) encodes glycosylated protein containing a C-terminal fibrinogen domain (Kim et al., 2000). Overexpression of ANGPTL4 promoted glucose uptake and glycolysis activity in colorectal cancer cells (Zheng et al., 2021). High ANGPTL4 expression was associated with pathological stage and shorter overall survival and disease-free survival in patients with breast cancer (Zhao et al., 2020a). INSR (Insulin Receptor) is a member of receptor tyrosine kinase which mediates the pleiotropic actions of insulin (Kadowaki et al., 1990). *NSR* rs11668724 G > A exhibited an increased pancreatic cancer risk (OR = 0.89, *p* = 4.21 × 10^−5^) (Zhao et al., 2020b). Upregulation of INSR promoted tumorigenesis and metastasis in tongue squamous cell carcinoma (Sun et al., 2018). The effects of insulin were used to enhance the therapeutic effectiveness of chemotherapeutic drugs through downregulation of INSR signaling (Agrawal et al., 2019). ADIPOQ (Adiponectin, C1Q And Collagen Domain Containing) is expressed in adipose tissue exclusively and is involved in metabolic and hormonal processes (Ferguson et al., 2010). ADIPOQ induced cytotoxic autophagy in breast cancer cells. The *ADIPOQ* rs266729 G/C polymorphism led to low expression levels of adiponectin in CRC. Decreased levels of adiponectin were regarded as risk factor for CRC in metabolic syndrome patients (Divella et al., 2017). ALDH1A3 (Aldehyde Dehydrogenase 1 Family Member A3) catalyzed the formation of retinoic acid and played roles in a diverse range of biological characteristics within cancer stem cells (Hsu et al., 1994; Duan et al., 2016). MMP1 (Matrix Metallopeptidase 1) influenced the progression of uveal melanoma from stage 3 to stage 4 and was correlated with OS and disease-free survival (Wang et al., 2021b). In future studies, it is warranted to investigate the biological functions of these genes in CRC.

There are some limitations in our study. First, there is no relevant experimental verification owing to lack of conditions in our study. All CRC patients were obtained from public datasets. Second, our risk model needs to be evaluated in clinical setting. Large-scale multi-center cohort will be explored in the predictive performance of the lipid metabolism-mediated signature for risk stratification. The prognostic role of the model will be further evaluated in patients with CRC.

## Conclusion

A valid and innovative 11-lipid metabolism gene signature model was constructed to predict the prognosis of CRC patients as an independent risk factors, including GGT5, ASAH1, HMGCL, CD36, DPM2, ACOX1, ANGPTL4, INSR, ADIPOQ, ALDH1A3, and MMP1. These dyregulated signature genes were involved in lipid metabolism pathway and Th1 and Th2 cell differentiation. Furthermore, our risk signature was correlated with high infiltration levels of T cells with better prognosis, including Th2 cells, Tregs, and Tgd cells.

## Data Availability

Publicly available datasets were analyzed in this study. The names of the repository/repositories and accession number(s) can be found in the article/Supplementary Material.

## Supplementary material

SUPPLEMENTARY FIGURE S1 Technical details of consensus molecular subtypes analysis. **(A)** consensus index. **(B)** *K* = 2 was regarded as the optimal value in consensus molecular subtypes of colorectal cancer. CDF: Cumulative Distribution Function.

SUPPLEMENTARY FIGURE S2 The association between risk score and clinical features, including age, **(A)** gender **(B)**, and race **(C)**. “≤60”: Patients less than or equal to 60 years of age; “>60”: Patients over 60 years of age.
