# Supplementary figures and images for: Identification of a novel lipid metabolism-related gene signature for predicting colorectal cancer survival
Source: Front Genet. 2022 Sep 6;13:989327. doi: 10.3389/fgene.2022.989327 (PMC9485806; doi:10.3389/fgene.2022.989327)

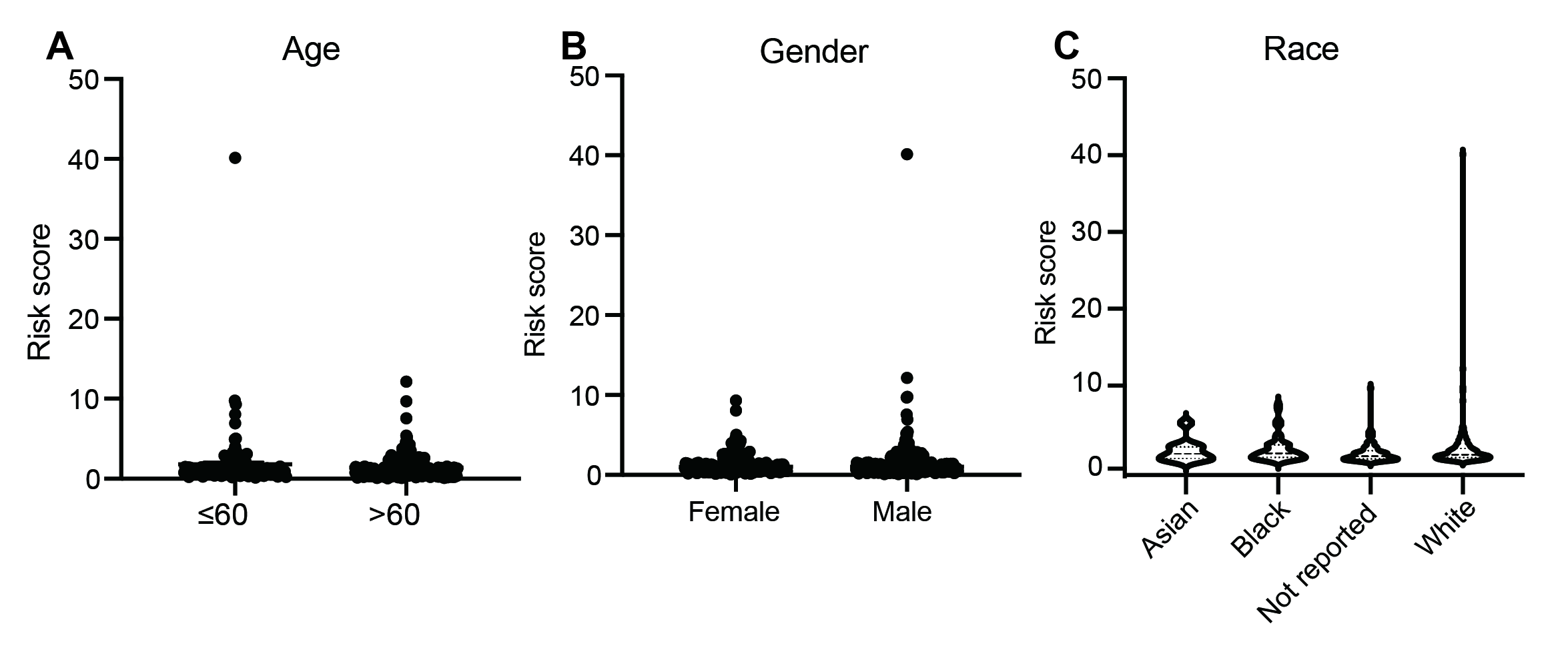

Supplement: Supplementary file 3 [file Image2.TIF]

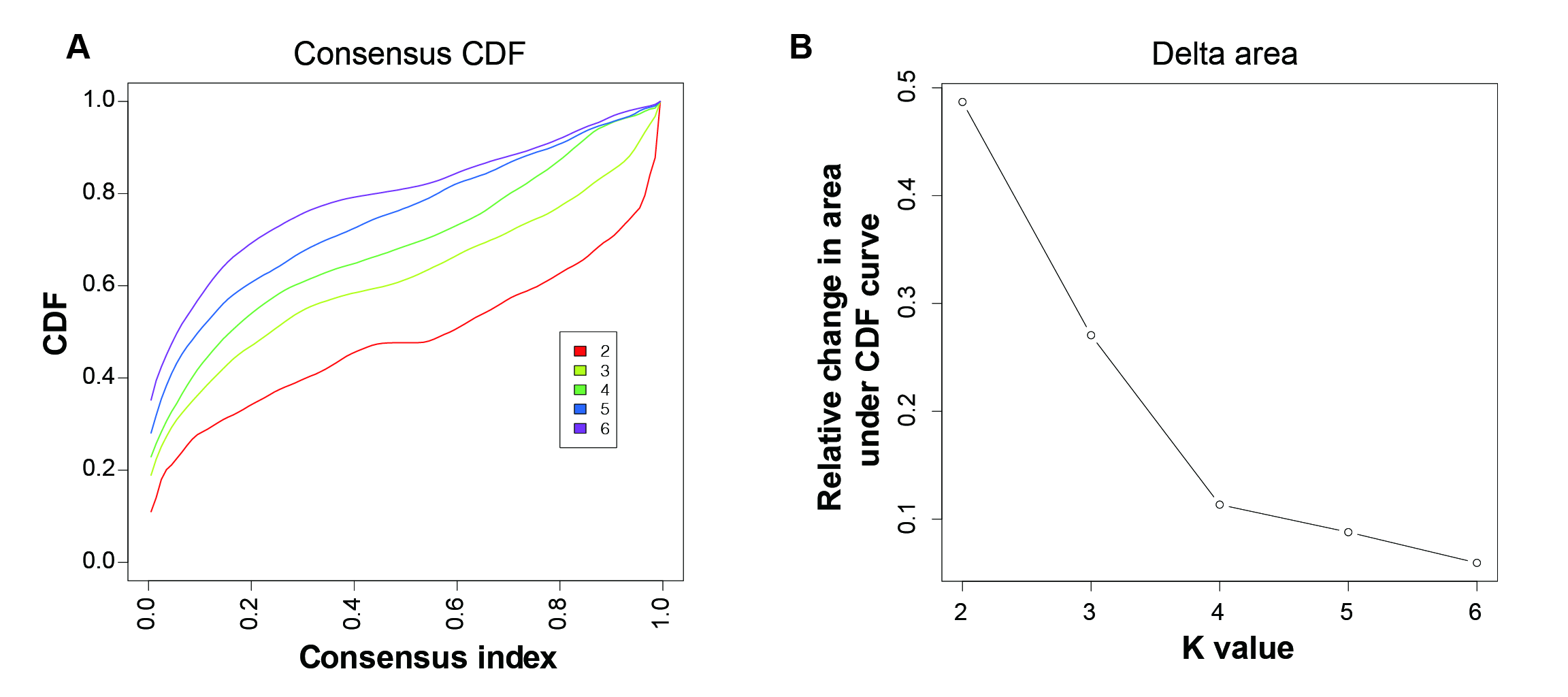

Supplement: Supplementary file 4 [file Image1.TIF]
